# Genome Sequence of “*Candidatus* Nitrosocosmicus franklandus” C13, a Terrestrial Ammonia-Oxidizing Archaeon

**DOI:** 10.1128/MRA.00435-19

**Published:** 2019-10-03

**Authors:** Graeme W. Nicol, Linda Hink, Cécile Gubry-Rangin, James I. Prosser, Laura E. Lehtovirta-Morley

**Affiliations:** a Laboratoire Ampère, École Centrale de Lyon, L’Université de Lyon, Écully, France; b School of Biological Sciences, University of Aberdeen, Aberdeen, United Kingdom; c School of Biological Sciences, University of East Anglia, Norwich, United Kingdom; Indiana University, Bloomington

## Abstract

“*Candidatus* Nitrosocosmicus franklandus” C13 is an ammonia-oxidizing archaeon (AOA) isolated from soil. Its complete genome is 2.84 Mb and possesses predicted AOA metabolic pathways for energy generation and carbon dioxide fixation but no typical surface layer (S-layer) proteins, only one ammonium transporter, and divergent A-type ATP synthase genes.

## ANNOUNCEMENT

Ammonia-oxidizing archaea (AOA) belonging to the phylum Thaumarchaeota play a central role in the global nitrogen cycle (1). The genus “*Candidatus* Nitrosocosmicus” is placed within the Nitrososphaerales, one of four orders of AOA (2, 3). “*Candidatus* Nitrosocosmicus” strains are widely distributed in soil (4) and have recently been cultivated from arable soil, coal-tar-contaminated sediment, and a wastewater treatment plant (5–7).

“*Candidatus* Nitrosocosmicus franklandus” C13 was isolated from a pH 7.5 arable soil sampled from an agricultural plot at the Scottish Rural College, Craibstone Estate, Aberdeen, United Kingdom (UK grid reference NJ872104) (5). An enrichment was obtained after inoculating soil (1% [wt/vol]) into inorganic freshwater medium often used for cultivating nonmarine AOA, with isolation achieved by the addition of antibiotics to eliminate contaminating bacteria. “*Ca.* N. franklandus” C13 grows optimally at 37°C and is routinely cultured with 2 mM ammonium chloride, with batch cultures typically taking approximately 10 days to reach stationary phase. Ammonia monooxygenase subunit A gene (*amoA*) and 16S rRNA gene sequences possessed 92 and 99% sequence similarity, respectively, to those of strain “*Ca.* Nitrosocosmicus oleophilus” (6), indicating taxonomic affiliation to the proposed genus “*Ca.* Nitrosocosmicus” but likely as a separate species (5).

DNA was extracted from pelleted cells using a standard SDS buffer and phenol:chloroform:isoamyl alcohol chemical lysis method (8) and sequenced using Illumina MiSeq and Oxford Nanopore MinION platforms. A library for paired-end sequencing on the Illumina MiSeq platform was prepared using a Nextera XT kit, according to the manufacturer’s instructions (Illumina, San Diego, CA, USA). Sequencing was performed with an Illumina MiSeq V2 kit, producing 11.9 million reads with an average length of 147 bp, with quality trimming performed using TrimGalore (https://github.com/FelixKrueger/TrimGalore). For MinION sequencing, DNA was sheared to approximately 8 kb using a g-TUBE (Covaris, Brighton, UK) and a library prepared with the SQK-MAP006 kit, according to the manufacturer’s instructions (Oxford Nanopore Technologies, Oxford, UK). Sequencing on a MinION flow cell produced 139,341 reads with an average length of 1,511 bp.

The genome was assembled using SPAdes (9) and Canu (10), using default settings for both MinION and Illumina data. For the MinION data, Nanopolish (https://github.com/jts/nanopolish) was used to improve the consensus sequence. The assembly resulted in three large contigs which were aligned against the closest reference genomes (those of “*Ca*. Nitrosocosmicus oleophilus” [6] and “*Ca.* Nitrosocosmicus exaquare” [7]) using Mauve (11). This allowed a prediction of the orientation of the three contigs, and gaps were subsequently closed by long-range PCR using the MasterAmp extra-long PCR kit (Epicentre, Madison, WI, USA) and Sanger sequencing.

The closed genome was annotated using the MaGe platform (12) and consists of 2,836,447 bases and a GC content of 34.07%. The genome has 3,180 protein-encoding sequences and two copies of the *rrn* operon. The ammonia monooxygenase (AMO) of AOA is likely encoded by four subunits (*amoA*, *amoB*, *amoC,* and a putative fourth subunit, *amoX*). The “*Ca.* N. franklandus” C13 genome contains one copy each of *amoA*, *amoB,* and *amoX* and three identical copies of *amoC*. While the *amoA* and *amoX* genes are adjacent, other *amo* subunits are dispersed throughout the genome, as is typically observed in Nitrososphaerales (13). Unusually, “*Ca.* N. franklandus” C13 and other “*Ca.* Nitrosocosmicus” genomes (6, 7) possess only one ammonium transporter gene (*amt*), in contrast to members of other thaumarchaeal genera, which possess at least two nonidentical *amt* genes.

Typical horseshoe-type tricarboxylic acid cycle genes previously reported in Thaumarchaeota are present in “*Ca.* N. franklandus” C13. The key genes for the 3-hydroxypropionate-4-hydroxybutyrate carbon fixation pathway were found, as expected, as “*Ca.* N. franklandus” C13 grows autotrophically on bicarbonate as a sole carbon source (14). All key genes of the nonoxidative pentose phosphate pathway are present but with a phosphogluconolactonase for the oxidative branch of the pentose phosphate pathway that lacks homology with non-“*Ca.* Nitrosocosmicus” AOA. Dehydrogenase, which was previously reported in “*Candidatus* Nitrososphaera gargensis,” could not be identified (13). “*Ca.* N. franklandus” C13 has the genetic potential to metabolize mannosylglycerate, which was previously reported for “*Ca*. N. gargensis” and may act as an osmolyte (13). Some subunits of the A-type ATP synthase are divergent from those found in other AOA, with the exception of those found in “*Candidatus* Nitrosotalea devanaterra” (9). “*Ca.* N. franklandus” C13 is nonmotile in laboratory culture and lacks genes for archaella. Genes falling into Clusters of Orthologous Genes (COG) category N (motility) are predicted to be involved in sensing rather than movement.

Surprisingly, the genome of “*Ca.* N. franklandus” C13, along with other characterized “*Ca.* Nitrosocosmicus” and “*Candidatus* Nitrosocaldus” genomes, lacks the main S-layer protein (encoded by *slp1*), which was previously considered to be the main component of the thaumarchaeal cell wall (15, 16). It was not possible to identify a potential alternative pathway for the S-layer protein within the genome of “*Ca.* N. franklandus” C13, but many cell wall-associated genes are putatively involved in amino sugar transformations, which warrants future investigation.

### Data availability.

The genome sequence was deposited in the European Nucleotide Archive under accession number LR216287. The raw sequencing reads are available in the NCBI Sequence Read Archive under accession number PRJNA530370.
